# Interaction of non-human primate complement and antibodies with hypermucoviscous *Klebsiella pneumoniae*

**DOI:** 10.1186/s13567-016-0325-1

**Published:** 2016-03-08

**Authors:** Esteban Soto, Sylvia Marchi, Amy Beierschmitt, Michael Kearney, Stewart Francis, Kimberly VanNess, Michel Vandenplas, MaryAnna Thrall, Roberta Palmour

**Affiliations:** Department of Epidemiology and Medicine, University of California, Davis-School of Veterinary Medicine, Davis, CA USA; Department of Biomedical Sciences, Ross University School of Veterinary Medicine, Basseterre, St. Kitts, West Indies; Behavioural Science Foundation, Estridge Estate, Basseterre, St. Kitts, West Indies; Department of Pathobiological Sciences, School of Veterinary Medicine, Louisiana State University, Baton Rouge, LA 70803 USA

## Abstract

Emergent hypermucoviscosity (HMV) phenotypes of *Klebsiella pneumoniae* have been associated with increased invasiveness and pathogenicity in primates. In this study, we investigated the interaction of African green monkeys (AGM) (*Chlorocebus aethiops sabaeus*) complement and antibody with HMV and non-HMV isolates as in vitro models of primate infection. Significantly greater survival of HMV isolates was evident after incubation in normal serum or whole blood (*p* < 0.05) of AGM donors when compared to non-HMV strains. Greater survival of HMV strains (*p* < 0.05) was found after incubation in whole blood and serum from seropositive donors when compared to seronegative donor samples. Additionally, significantly greater amounts of *K. pneumoniae* were phagocytozed by AGM leukocytes when complement was active (*p* < 0.05), but no difference in uptake was observed when serum from seropositive or seronegative animals was used in challenged cells utilizing flow cytometry. Results demonstrate that interaction of cellular and humoral immune elements play a role in the in vitro killing of *K. pneumoniae*, particularly HMV isolates. Neither AGM serum, nor washed whole blood effectively killed HMV isolates; however, assays using heparinized whole blood of seronegative donors significantly reduced viability of HMV and non-HMV strains. The lack of bacterial killing observed in seropositive donors treatments could be at least partially associated with low IgG2 present in these animals. A better understanding of the pathogenesis of klebsiellosis in primates and host immune response is necessary to identify surface molecules that can induce both opsonizing and bactericidal antibody facilitating killing of *Klebsiella*, and the development of vaccines in human and animals.

## Introduction

*Klebsiella pneumoniae* is a Gram-negative bacterium member of the family Enterobactericeae typically found in the environment and on mucosal surfaces. In both human and veterinary medicine this bacterium is regarded as an emergent and common nosocomial pathogen associated with a range of infections [1–6].

A novel, invasive form of *K. pneumoniae*, referred to as hypervirulent and typically presenting a hypermucoviscous (HMV) phenotype, has emerged over the last two decades [7–10]. Distinct capsular components and an increased amount of capsular material in HMV *K. pneumoniae* have been described. The HMV strains have been associated with serotypes K1–K6 in humans, non-human primates, and marine mammals, and have been associated more frequently with osteomyelitis, endophthalmitis, meningitis, severe respiratory infection related with suppurative pneumonia, pleuritis, and multi systemic abscesses in the lung, liver, cervix, brain and septicemia; however lack of specific clinical presentation in klebsiellosis makes premorbid diagnosis difficult [5, 11–14]. Additionally, HMV isolates are associated with several virulence genes including *rmpA* (regulator of mucoid phenotype) and *magA* (mucoviscosity-associated) [7, 15]. The *magA* gene encodes a 43-kD outer membrane protein, whereas the *rmpA* gene is a transcriptional activator of colanic acid biosynthesis [16].

Although *K. pneumoniae* has been recognized as an important nosocomial infection, and HMV isolates are often associated with high morbidity and mortality in a wide range of mammals, the pathogenesis of the disease and the epizootiology of the pathogen remain poorly characterized. Additionally, little work elucidating the role of the HMV phenotype in *K. pneumoniae* pathogenicity exists, no vaccines are available, and few studies provide direct comparison of HMV and non-HMV isolates. Recently, isolates recovered from African Green monkeys (AGM) presenting with a HMV phenotype, and belonging to serotype K1 and K5 were found to be significantly more virulent and resistant than non-HMV isolates in in vitro, serum, and oxidative-mediated killing assays [17].

To gain a better understanding about the pathogenesis of this important emergent disease in primates, and to investigate the role of innate and adaptive immune components in the protection against *K. pneumoniae*, we evaluated the role of complement and antibody in protection against HMV and non-HMV isolates using the AGM. An understanding of the relevant protective immune mechanisms against klebsiellosis is essential if a vaccine is to be developed in a timely manner.

## Materials and methods

### Animal subjects and inclusion criteria

Both the Ross University School of Veterinary Medicine and Behavioural Science Foundation Institutional Animal Care and Use Committees reviewed and approved this study. In order to assess prior exposure or current infection (clinical or sub-clinical) of *K. pneumoniae*, 25 female captive AGM, ages 5–10 years old from the Behavioural Science Foundation, St. Kitts, were screened serologically and molecularly following protocols by Cox et al. [17] (Table 2).

In order to collect blood and swabs from the AGM, animals were isolated by tunneling into a squeeze cage and anesthetized with ketamine (10 mg/kg intramuscular) and brought to a central husbandry area for examination by a veterinarian. All of these animals were confirmed as healthy on physical examination. Depending on the experiment, whole blood (4–6 mL/animal) was collected from the femoral vein using red top, EDTA or sodium heparin vacutainer tubes (Becton Dickinson and Company, Sparks, MD, USA), and placed on ice. Six animals (three seropositive and three seronegative donors) were chosen for further investigation. They were bled three additional times giving them at least 2 weeks between sample collections. Animals recovered from anesthesia in their home cages or tunnels of their home cages. They were kept sheltered and under observation until completely recovered.

Nasal, vaginal, fecal and oral swabs were collected from the AGM and suspended in 500 μL PBS and total DNA was extracted utilizing, the DNeasy Kit (nasal, vaginal and oral) or the QIAamp DNA Stool Mini Kit (fecal) (Qiagen, Valencia, CA, USA). Extracted DNA served as a template in a real-time PCR assay for the detection of the *K. pneumoniaekhe*, *rpmA*, or *magA* genes (Table 2) following published protocols [18]. Blood collected from donor animals was subjected to complete blood counts and biochemical analysis of plasma using Abaxis HM5c Hematology Analyzer and Abaxis VetScan VS2 (Abaxis North America, Union City, CA, USA). Additionally, protein electrophoresis analysis of serum was performed at Kansas State University Veterinary Diagnostic Laboratory using the TITAN GEL Serum Protein System (Helena Laboratories, Beaumont, TX, USA).

### Bacterial strains and culture conditions

*Klebsiella pneumoniae* strains cultured from AGM with single or multifocal abscesses were isolated at the Ross University School of Veterinary Medicine Diagnostic Laboratory from 2010 to 2012. Identification and characterization of the isolates was made according to standard clinical microbiologic and molecular methods (Table 1) [6, 17, 18]. For general use, *K. pneumoniae* was grown on 5% sheep blood agar plates, brain–heart infusion broth (BHI) or Luria–Bertani (LB) broth (Sigma-Aldrich, St. Louis, MO, USA) at 37 °C. The mucoviscosity levels were determined by string test and centrifugation (Table 1) [19, 20]. Briefly, *K. pneumoniae* isolates were cultivated at 37 °C overnight. The following morning 1.2 mL of optical density (OD)_600_ normalized bacteria grown in LB broth was centrifuged in microcentrifuge tubes at 2000 *g* for 5 min. The absorbance of the supernatant was measured at OD_600_. A representative K1, K5 and non-HMV isolate previously characterized were used for in vitro challenges [17].

**Table 1:** ***Klebsiella pneumoniae*** **isolates used in this study**

Isolate designation	Species identification	Host isolated from	HMV phenotype as determined by string test^a^	Detection of *khe* ^b^	Detection of *rmpA* ^b^	Detection of m*agA* ^b^	Serotype^c^
K1	*Klebsiella* *pneumoniae*	AGM	Positive (+++)	Positive	Positive	Positive	K1
K5	*Klebsiella* *pneumoniae*	AGM	Positive (++)	Positive	Positive	Negative	K5
NHM	*Klebsiella* *pneumoniae*	AGM	Negative	Positive	Negative	Negative	ND

AGM: African green monkey, ND: not determined.

^a^Done as described in [19, 20].

^b^Done as described in [18].

^c^Done as described in [15].

### Serologic assays for anti-*K. pneumoniae* immunoglobulins

Indirect ELISA was used to determine AGM IgG and IgM antibody concentrations against HMV and non-HMV-*K. pneumoniae* in serum from seropositive and seronegative donors. Protocols described by Cox et al. [17] were followed with modifications. Briefly, BD Falcon 96-well black/clear flat-bottom microtitre plates (Becton Dickinson and Company, Sparks, MD, USA) were coated with 5 × 10^6^ colony forming units (CFU) per well live *K. pneumoniae* in carbonate coating buffer, pH 9.6, at 100 µL per well, and incubated overnight at 4 °C. Plates were washed three times in PBS containing 0.05% Tween-20 (PBST), and blocked for 1 h at room temperature (RT) with ELISA Blocking Buffer (Sigma-Aldrich, St. Louis, MO, USA). Serum samples were diluted 1:50 in PBST. Negative control wells were incubated with PBST alone. Plates were incubated overnight at 4 °C and washed 5× with PBST. Rabbit polyclonal to Human IgG-FITC, or Rabbit Anti-Human IgM H&L-FITC secondary antibodies (Abcam, Cambridge, MA, USA) were diluted in PBST following manufacturer recommendations, and 100 µL were added to each well. After incubation at room temperature for 2 h, the plate was washed 5× with PBST before adding 100 µL of PBST. Fluorescence at excitation of 493 nm and emission of 528 nm was recorded using the Infinite M200 96-well-plate reader (Tecan Group Ltd., Mannedorf, Switzerland).

Quantification of IgG sub-types in donor samples was performed using PeliClass human IgG subclass kit following manufacturer’s instructions (Sanquin Reagents, Amsterdam, The Netherlands).

### Serologic assays for *K. pneumoniae* complement deposition

Indirect ELISA was used to compare complement C3/C3b and C5–9 (membrane attack complex) deposition on *K. pneumoniae* using serum from seropositive and seronegative donors. Protocols described by Cox et al. [17] were followed with modifications. Seeding of antigen was done as previously described. After overnight incubation at 4 °C, wells were washed 5× with PBST. Mouse monoclonal to C3/C3b, or Mouse monoclonal to C5b-9 secondary antibody (Abcam, Cambridge, MA, USA) were diluted in PBST following manufacturer recommendations, and 100 µL were added to each well. Plates were incubated at RT for 2 h, followed by 5× washes with PBST before adding Rat monoclonal to IgG2a HRP (Abcam) as tertiary antibody. After incubation at 25 °C for 1 h, the plate was washed 5× with PBST before adding 100 µL of ABTS Peroxidase Substrate (KPL, Gaithersburg, MD, USA) to each well. The ELISA reaction was stopped after 30 min with 100 µL 1% sodium dodecyl sulfate, and the optical density (OD) of the reactions was read at 405 nm with a SpectraMax M2/M2e Microplate Reader (Molecular Devices, Sunnyvale, CA, USA).

### *Klebsiella* serum and whole blood killing assays

Whole blood and serum bactericidal assays developed by MacLennan et al. [21] were modified to investigate in vitro killing of serum sensitive *E. coli*, HMV and non-HMV *K. pneumoniae* isolates. Blood or serum from three seropositive and three seronegative donors was used (Table 2). Killing of different bacterial isolates was assessed using (a) heparinized blood (4 IU/mL sodium heparin) within 2 h of venesection; (b) serum separated within 2 h and stored at –80 °C; (c) heat inactivated serum (inactivated by heating serum to 56 °C for 30 min); or (d) blood-cell-suspensions, prepared by washing blood twice with Roswell Park Memorial Institute (RPMI)-1640 medium (Gibco by Life Technologies, Grand Island, NY, USA) to remove antibody and complement before resuspending in RPMI to the original blood volume (whole blood-RPMI). Viable bacteria (2 × 10^5^ in 20 μL), were added to 180 μL of 100% whole blood, undiluted serum (normal and heat-inactivated), or blood-cell-suspensions to give a final bacterial concentration of 1 × 10^6^/mL regardless of white cell count. These mixtures were then incubated at 37 °C on a rocker plate at 20 rpm. Numbers of viable bacteria were determined after 60 min by serial dilution of 20 μL of the mixture plated in triplicate on blood agar plates.

**Table 2:** **Inclusion criteria for African Green monkey donors in this study**

Donor	Age (years)	Sex	Anti-*Klebsiella pneumoniae* IgG titers^a^			Isolation of *K. pneumoniae* or detection of *khe*, *rmpA* or *magA* related genes^b^				Donor designation
		Female	K1	K5	Non-HMV	Fecal	Oral	Vaginal	Nasal	
1	11	Female	8192	8192	8192	–	–	–	–	Seropositive
2	5	Female	8192	8192	2048	–	–	–	–	Seropositive
3	5	Female	16 384	8192	8192	–	–	–	–	Seropositive
4	5	Female	1024	1024	1024	–	–	–	–	Seronegative
5	5	Female	512	256	512	–	–	–	–	Seronegative
6	6	Female	1024	1024	1024	–	–	–	–	Seronegative

^a^According to [17].

^b^According to [18].

### Phagocytosis by peripheral blood mononuclear and polymorphonuclear leukocytes assays

Within 1 h of collection, peripheral blood mononuclear cells (PBMC) and polymorphonuclear leukocytes (PMNL) were purified from blood collected in EDTA using Histopaque 1077 (Sigma-Aldrich, St. Louis, MO, USA) following manufacturer’s instructions and published protocols [17]. Erythrocytes were removed by hypotonic lysis in water for 30 s and isotonic conditions restored immediately by adding an equal volume of 2 × PBS. Thereafter the PMNL were washed twice in PBS and collected by low speed centrifugation. Viability of PMNL and PBMC was verified using trypan blue exclusion assays. PMNL and PBMC were plated at a concentration 1 × 10^6^ per well in 24-well tissue culture plates (Becton Dickinson and Company, Sparks, MD, USA). After a 2 h incubation at 37 °C, cells were challenged with a suspension of bacteria in cell culture medium containing 10% serum (normal or heat inactivated from seropositive or seronegative donors) to obtain an multiplicity of infection (MOI) of 1:20 (leukocyte to bacteria). One hour post-incubation, wells were washed with PBS and incubated for 1 h with fresh medium containing gentamicin (100 µg/mL) (Sigma-Aldrich, St. Louis, MO, USA) to kill extracellular bacteria, followed by three washes with PBS, and later lysis by incubation in PBS supplemented with 0.5% Triton X-100 (Sigma-Aldrich). Internalized bacteria were quantified by plating appropriate dilutions on agar plates.

### Oxidative burst assay

The Bursttest assay (Becton Dickinson) and protocols by Gondwe et al. [22] and Lin et al. [23] were used to measure oxidative burst activity in AGM PMNL and PBMC by flow cytometry by determining oxidation of dihydrorhodamine to rhodamine. Opsonization was by incubating 10^9^/mL of heat-killed *K. pneumoniae* in serum obtained from seropositive and seronegative donors for 1 h at room temperature at a ratio of 1:10. RPMI washed cells were stimulated with opsonized heat-killed *K. pneumoniae* at 2 × 10^8^/mL for 10 and 60 min at 37 °C according to the manufacturer’s instructions before incubating with dihydrorhodamine. Following red cell lysis and fixation, leukocytes were analyzed on a FACSCalibur flow cytometer (Becton Dickinson). PMNL and PBMC were gated according to light scatter characteristics. Rhodamine fluoresces in the FL1 channel and oxidative burst activity was measured according to the manufacturer’s instructions as geometric mean fluorescence intensity from the FL1 histogram (arbitrary units). Controls consisted of PMA, fMLP, pre-opsonized *E. coli* and TLR-2 (PAM_3_CSK_4_) stimulation.

### Statistical analysis

The SAS^®^ (version 9.4, SAS Institute, Cary, NC, USA) GLM procedure was used to conduct an analysis of variance in a factorial arrangement of treatments. When overall significance was found post hoc comparisons were conducted with pairwise t tests of least-squares means. All tests were considered significant at *p* ≤ 0.05.

#### Serum and whole blood killing assays

Effects in the model included isolate (K1, K5, NHM, *E. coli*), treatment (whole blood cells, whole blood-RPMI, normal serum, heat-inactivated serum) and titer of donor (seropositive or seronegative) and the two-way and three-way interactions.

#### AGM PMNL and PBMC uptake of *Klebsiella pneumoniae*

Effects in the model included isolate (K1, K5, NHM), treatment (normal serum, heat-inactivated serum) and titer of donor (seropositive or seronegative) and the two-way and three-way interactions.

#### Immonuglobulin IgG and IgM and complelement proteins C3/C3b and C5-9 binding to *Klebsiella pneumoniae*

Effects in the model included isolate (K1, K5, NHM), and titer of donor (seropositive or seronegative) and the two-way interaction.

#### Immonuglobulin IgG subtype quantification in serum from seropositive and seronegative AGM donors

Two-sample *t* tests assuming equal variance were used to statistically compare IgG1, IgG2, IgG3 and IgG4 levels in seropositive and seronegative donors. *P* ≤ 0.05 was considered significant.

## Results

### Serologic and molecular diagnosis of klebsiellosis in African green monkey donors

**Figure 1:**
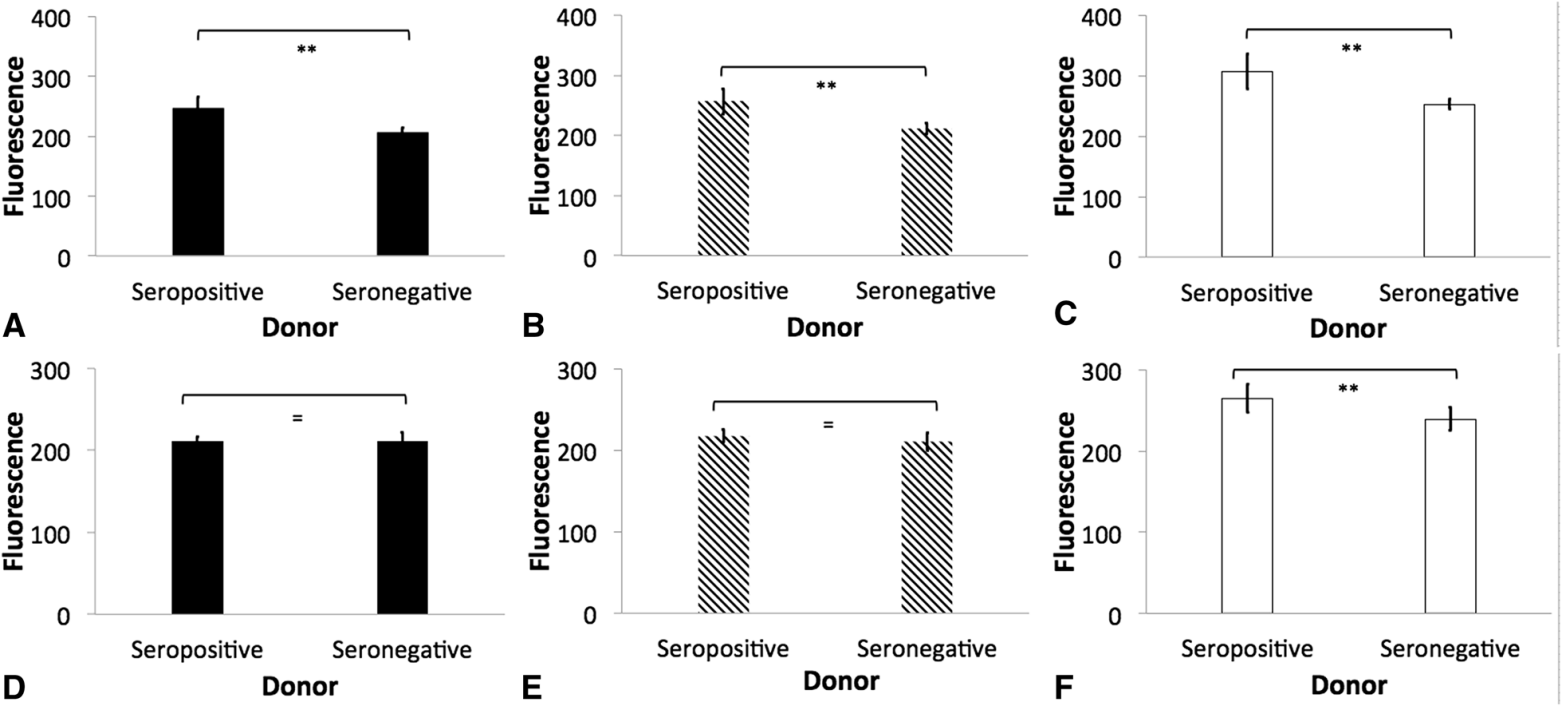
**Indirect ELISA demonstrating antibody binding to** ***Klebsiella pneumoniae.*** Binding of IgG (**A**, **B** and **C**) and IgM (**D**, **E**, **F**) to hypermucoviscous isolates K1 (**A** and **D**, black bars), K5 (**B** and **E**, diagonal lines) and non- hypermucoviscous (NHM) (**C** and **F**, white bars) *Klebsiella pneumoniae* using serum obtained from seropositive and seronegative African green was measured using the Infinite M200 96-well-plate reader (Tecan Group Ltd., Mannedorf, Switzerland). Fluorescence was recorded at excitation of 493 nm and emission of 528 nm. The error bars represent standard errors for triplicate samples, and the results shown are representative of three independent experiments. The error bars represent standard errors for triplicate samples, and the results shown are representative of three independent experiments. Asterisk indicates significant difference between treatments, *p* ≤ 0.05; Double asterisk indicates significant difference between treatments, *p* ≤ 0.001; Equal to indicates similarity between treatments, *p* > 0.05.

Real-time amplification of oral, nasal, vaginal and fecal swab material did not detect the *khe, magA,* or *rmpA* gene in any of the sampled monkeys. Three animals were classified as seropositive donors based on IgG titers >8192 against *K. pneumoniae* isolates K1 and K5 (Table 2; Figure 1). On the other hand, three seronegative donors were included presenting IgG titres of ≤ 1024 (Table 2; Figure 1). Similar amounts of anti-*K. pneumoniae* K1 and K5 IgM were detected in seropositive and seronegative donors (Figure 1). Significantly greater amount of anti-*K. pneumoniae* IgG and IgM binding to non-HMV isolates was detected when compared to those binding to K1 or K5 isolates. Complete blood counts, serum clinical chemistries, and protein electrophoresis of serum resulted in similar values between seropositive and seronegative donors (data not shown).

### IgG quantification in donor monkeys

**Figure 2:**
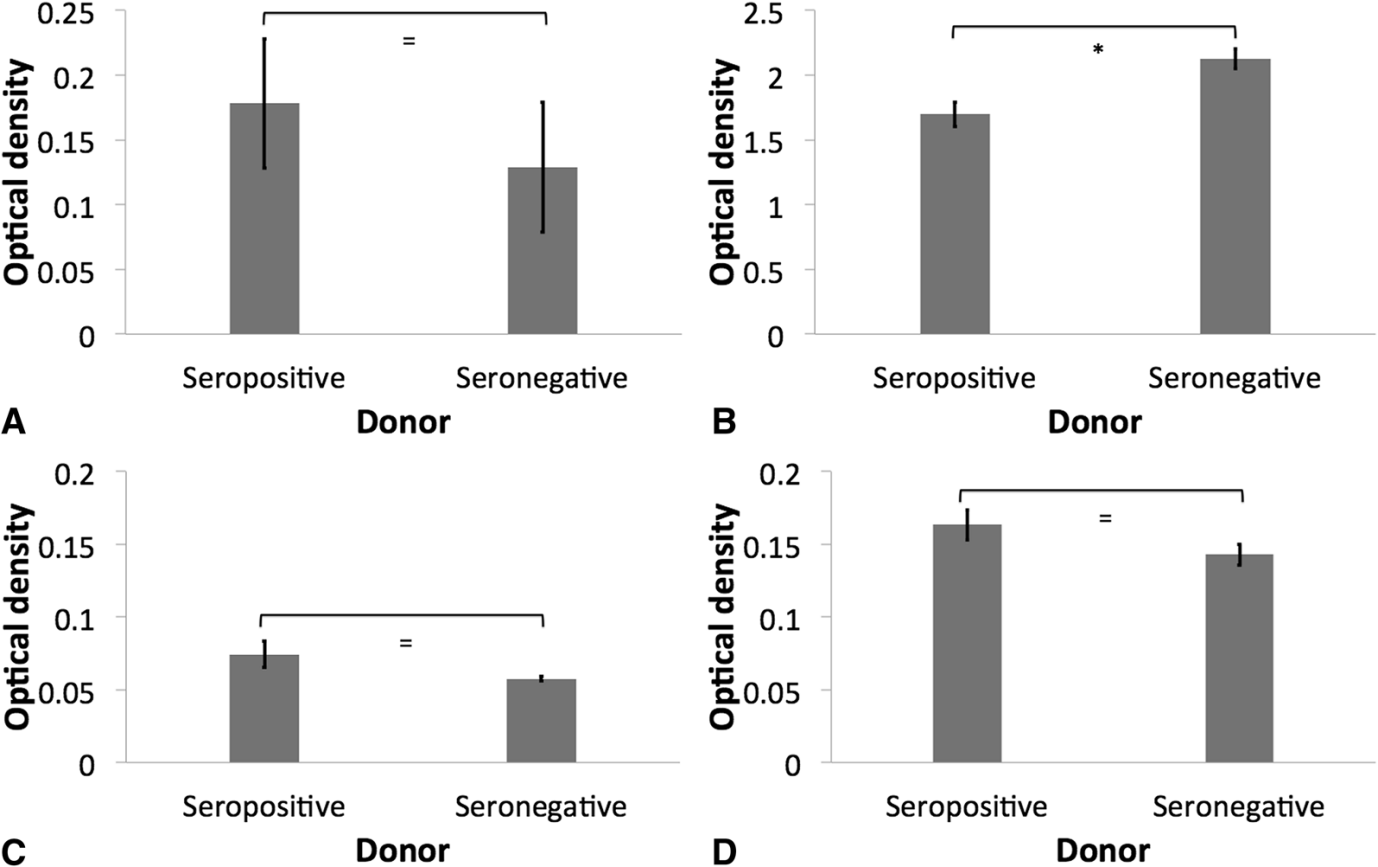
**Quantification of IgG sub-types in serum obtained from seropositive** **and seronegative African green monkey donors.** IgG1 (**A**), IgG2 (**B**), IgG3 (**C**) and IgG4 (**D**) in donor serum were quantified using PeliClass human IgG subclass kit following manufacturer’s instructions (Sanquin Reagents, Amsterdam, The Netherlands). Optical density of the reactions was read with a SpectraMax M2/M2e Microplate Reader (Molecular Devices, Sunnyvale, CA, USA). The error bars represent standard errors for triplicate samples, and the results shown are representative of three independent experiments. The error bars represent standard errors for triplicate samples, and the results shown are representative of three independent experiments. Asterisk indicates significant difference between treatments, *p* ≤ 0.05; Double asterisk indicates significant difference between treatments, *p* ≤ 0.001; Equal to indicates similarity between treatments, *p* > 0.05.

Concentrations of IgG1, IgG3 and IgG4 were similar in seropositive and seronegative donors (*p* < 0.05); however, levels of IgG2 antibodies were significantly lower in the seropositive donors (*p* = 0.004) (Figure 2).

### Complement binding to opsonized *K. pneumoniae*

**Figure 3:**
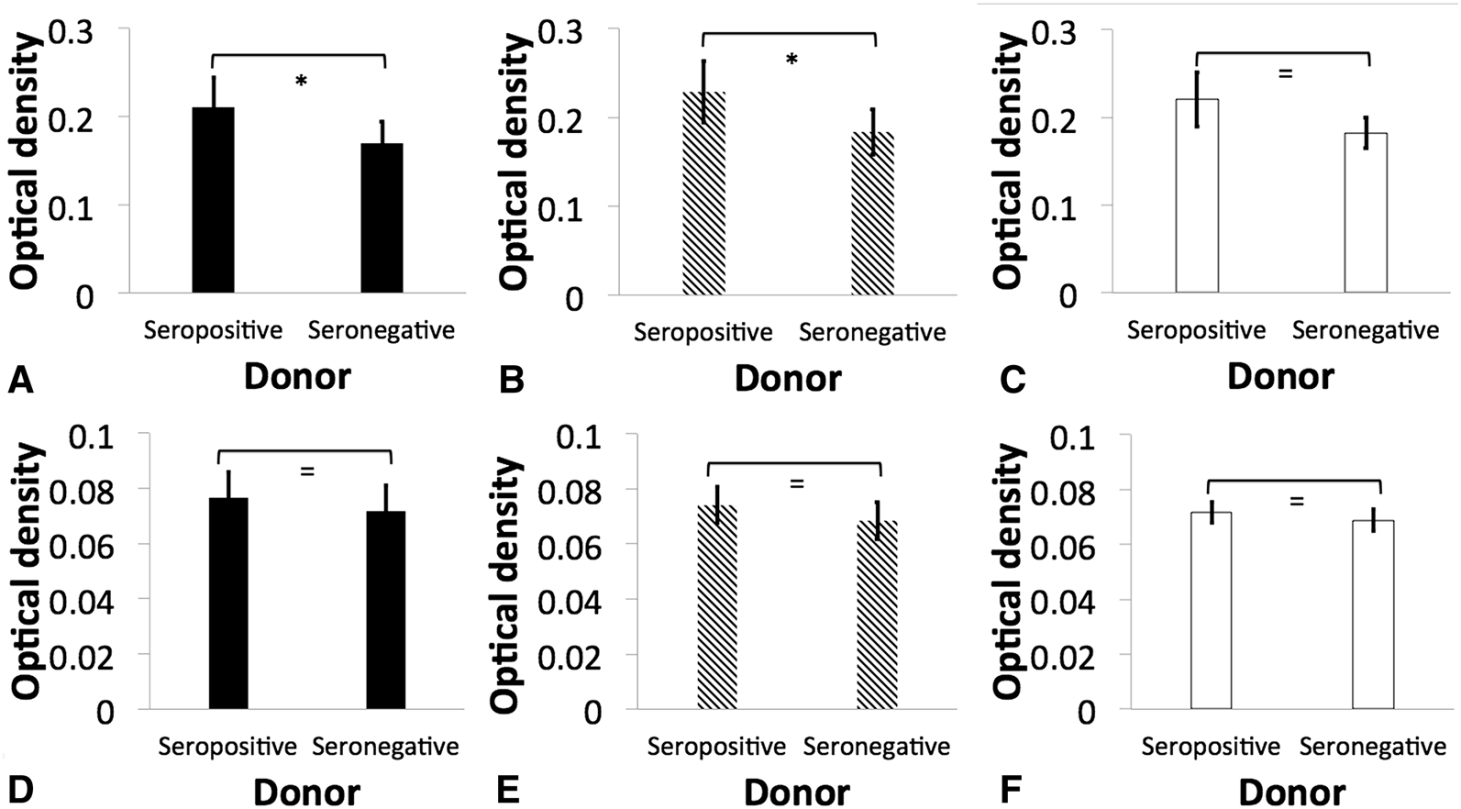
**Indirect ELISA demonstrating complement binding to** ***Klebsiella pneumoniae***. Binding of C3/C3b (**A**, **B** and **C**) and C5–9 (**D**, **E** and **F**) to hypermucoviscous isolates K1 (**A** and **D**, black bars), K5 (**B** and **E**, diagonal lines), and non- hypermucoviscous (NHM) (**C** and **F**, white bars) *Klebsiella pneumoniae* using serum obtained from seropositive and seronegative African green monkey was estimated by optical density measured with a SpectraMax M2/M2e Microplate Reader (Molecular Devices, Sunnyvale, CA, USA). The error bars represent standard errors for triplicate samples, and the results shown are representative of three independent experiments. Asterisk indicates significant difference between treatments, *p* ≤ 0.05; Double asterisk indicates significant difference between treatments, *p* ≤ 0.001; Equal to indicates similarity between treatments, *p* > 0.05.

Greater binding of C3/C3b to K1 and K5 *K. pneumoniae* was found in serum from seropositive donors than in serum from seronegative donors (*p* < 0.05) (Figure 3). There was similar binding of C3/C3b to NHM, K1 and K5 isolates (*p* > 0.05). Additionally, similar binding of C5–9 was detected when using serum of seropositive and seronegative donors, and in NHM, K1 and K5 isolates (*p* > 0.05) (Figure 3).

### Blood and serum killing of *K. pneumoniae* isolates

**Figure 4:**
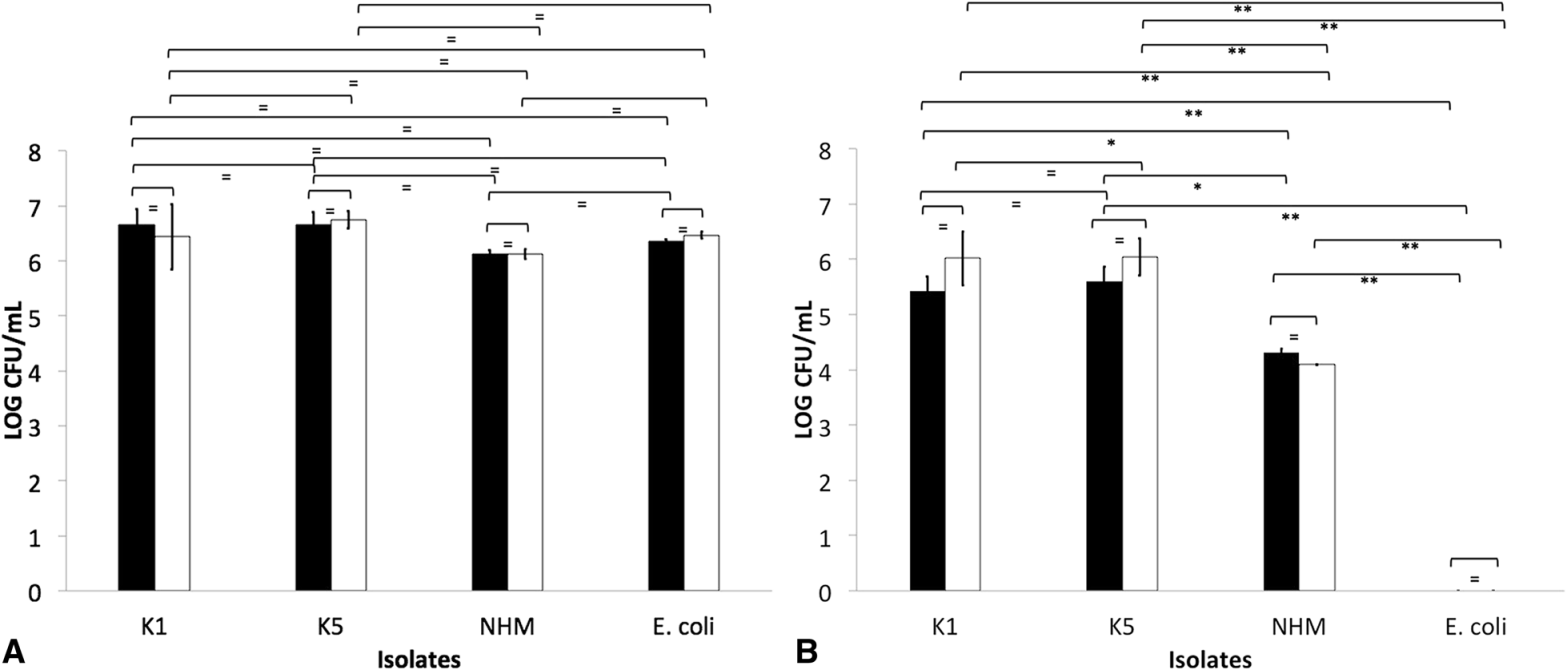
**Serum-mediated killing of isolates K1, K5 and non-** **hypermucoviscous (NHM)** ***Klebsiella pneumoniae*** **and** ***Escherichia coli.*** LOG CFU/mL of bacteria 1 h post-incubation in heat-inactivated serum (**A**) or normal serum (**B**) recovered from seronegative (dark bars) or seropositive (clear bars) African Green monkey. The error bars represent standard errors for triplicate samples, and the results shown are representative of three independent experiments. The error bars represent standard errors for triplicate samples, and the results shown are representative of three independent experiments. Asterisk indicates significant difference between treatments, *p* ≤ 0.05; Double asterisk indicates significant difference between treatments, *p* ≤ 0.001; Equal to indicates similarity between treatments, *p* > 0.05.

Similar survival was detected in serum-sensitive *E. coli*, NHM, K1 and K5 *K. pneumoniae* isolates when incubated in heat-inactivated serum from seropositive and seronegative donors (*p* > 0.05) (Figure 4A). There was 100% survival in all treatments. K1 and K5 *K. pneumoniae* presented significantly greater survival than non-HMV isolates when challenged with normal serum collected from seropositive and seronegative donors (*p* < 0.05) (Figure 4B). Although not significant (*p* > 0.05), there was greater survival of K1 and K5 *K. pneumoniae* isolates in normal serum from seropositive, as compared to seronegative donors. No difference in survival was detected between K1 and K5 *K. pneumoniae* isolates (*p* > 0.05). Additionally, *E. coli* was significantly more susceptible than HMV and non-HMV *K. pneumoniae* isolates (*p* < 0.05) (Figure 4B).

**Figure 5:**
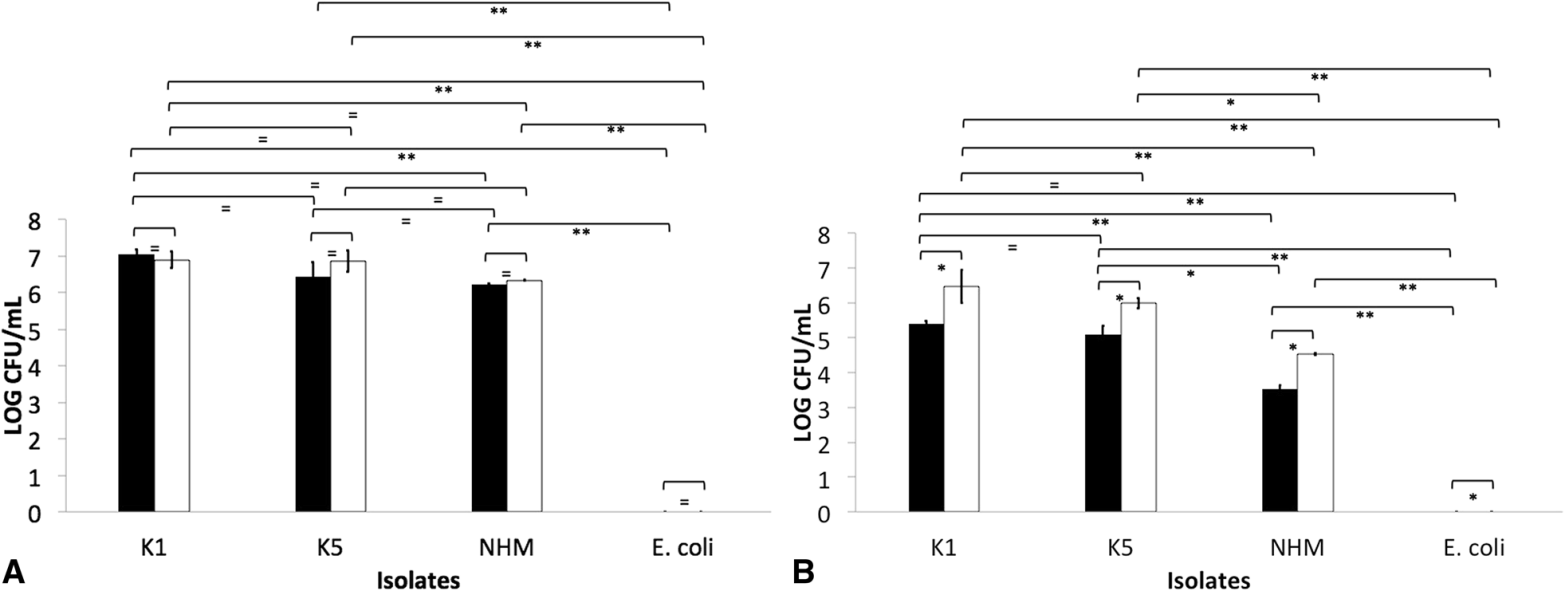
**Whole blood-mediated killing of isolates K1, K5 and non-** **hypermucoviscous (NHM)** ***Klebsiella pneumoniae*** **and** ***Escherichia coli.*** LOG CFU/mL of bacteria 1 h post-incubation in RPMI washed whole blood (**A**) or heparinized-whole blood (**B**) recovered from seronegative (dark bars) or seropositive (clear bars) African Green monkey. The error bars represent standard errors for triplicate samples, and the results shown are representative of three independent experiments. Treatments with different letters are significantly different from one another at *p* < 0.05. Asterisk indicates significant difference between treatments, *p* ≤ 0.05; Double asterisk indicates significant difference between treatments, *p* ≤ 0.001; Equal to indicates similarity between treatments, *p* > 0.05.

When incubated in whole blood-RPMI collected from seropositive or seronegative donors, similar survival was detected in NHM, K1 and K5 *K. pneumoniae* (*p* > 0.05); however, all serum-sensitive *E. coli* were killed (Figure 5A). Significantly greater survival of K1, K5 and NHM *K. pneumoniae* isolates was detected after incubation in heparinized blood from seropositive donors as compared to that from seronegative donors (*p* < 0.05) (Figure 5B). Significantly greater survival was detected in HMV isolates (K1 and K5) than in non-HMV *K. pneumoniae* isolates (*p* < 0.05). All *E. coli* was killed (Figure 5B).

### Uptake of opsonized *K. pneumoniae* by AGM leukocytes

**Figure 6:**
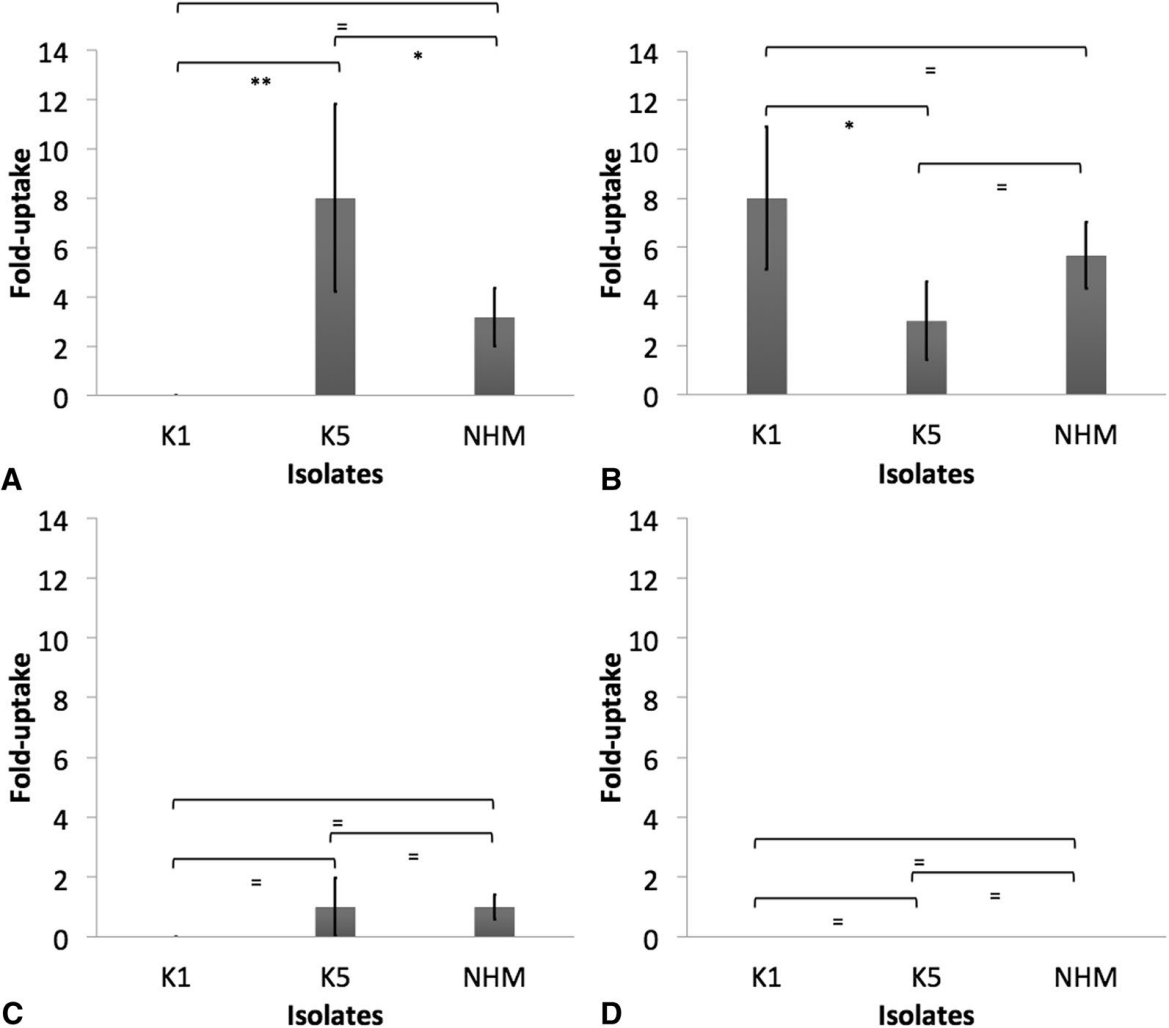
**Peripheral blood mononuclear cells (PBMC) uptake of** ***Klebsiella pneumoniae.*** Uptake of opsonized K1, K5, and non-HMV *K. pneumoniae* with normal serum (**A** and **B**) or heat-inactivated serum (**C** and **D**) from seropositive (**A** and **C**) or seronegative (**B** and **D**) donors by African Green monkey (AGM) PBMC was measured in vitro as described. The results are expressed as fold-uptake when compared to PBS opsonized bacteria. The error bars represent standard errors for triplicate samples, and the results shown are representative of three independent experiments. Asterisk indicates significant difference between treatments, *p* ≤ 0.05; Double asterisk indicates significant difference between treatments, *p* ≤ 0.001; Equal to indicates similarity between treatments, *p* > 0.05.

**Figure 7:**
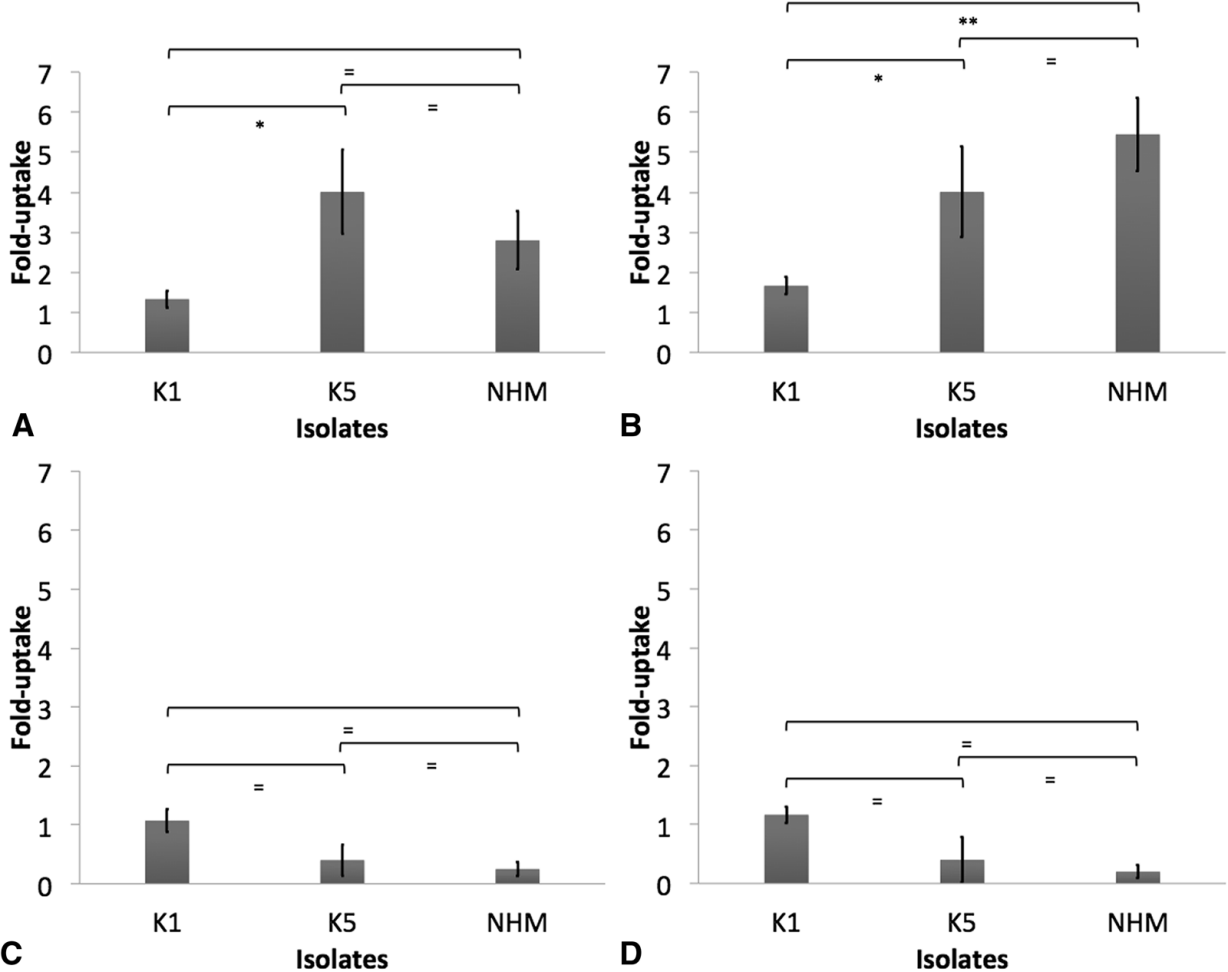
**Polymorphonuclear leukocytes (PMNL) uptake of** ***Klebsiella pneumoniae.*** Uptake of opsonized K1, K5, and non-HMV *K. pneumoniae* with normal serum (**A** and **B**) or heat-inactivated serum (**C** and **D**) from seropositive (**A** and **C**) or seronegative (**B** and **D**) donors by African Green monkey (AGM) PMNL was measured in vitro as described. The results are expressed as fold-uptake when compared to PBS opsonized bacteria. The error bars represent standard errors for triplicate samples, and the results shown are representative of three independent experiments. Asterisk indicates significant difference between treatments, *p* ≤ 0.05; Double asterisk indicates significant difference between treatments, *p* ≤ 0.001; Equal to indicates similarity between treatments, *p* > 0.05.

**Figure 8:**
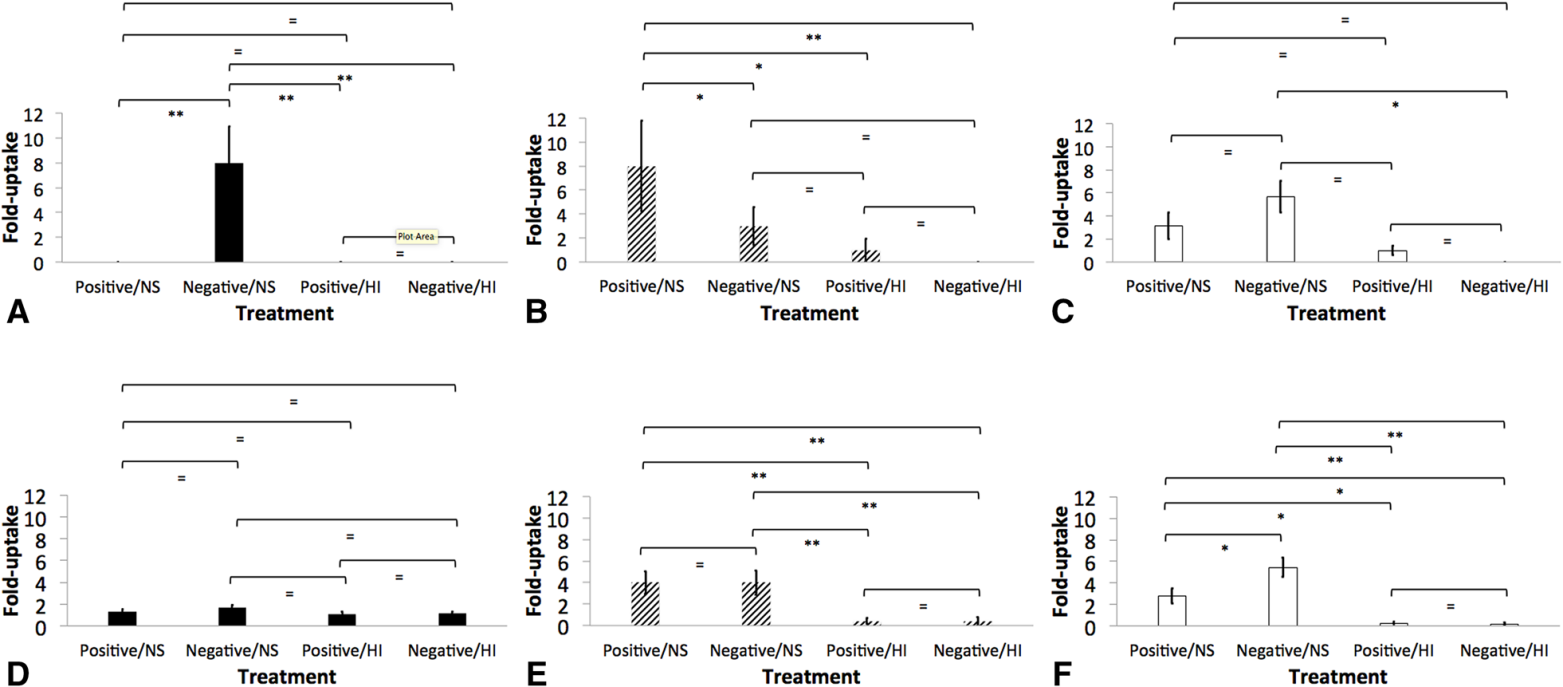
**Role of complement and antibodies as opsonins of** ***Klebsiella pneumoniae*** **in in vitro assays.** Uptake of hypermucoviscous K1 (**A** and **D**, black bars), K5 (**B** and **E**, diagonal bars), and non-HMV *K. pneumoniae* (**C** and **F**, white bars) by African Green monkey (AGM) peripheral blood mononuclear cells (PBMC) (**A**, **B** and **C**) or polymorphonuclear leukocytes (PMNL) (**D**, **E** and **F**) was determined as described. The results are expressed as fold-uptake of opsonized bacteria with normal serum from seropositive (Positive/NS) or seronegative (Negative/NS) donors, or heat-inactivated serum from seropositive (Positive/HI) or seronegative (Negative/HI) donors when compared to PBS opsonized bacteria. The error bars represent standard errors for triplicate samples, and the results shown are representative of three independent experiments. Asterisk indicates significant difference between treatments, *p* ≤ 0.05; Double asterisk indicates significant difference between treatments, *p* ≤ 0.001; Equal to indicates similarity between treatments, *p* > 0.05.

AGM PMBC and PMNL phagocytized significantly greater amounts of K1, K5 and NHM *K. pneumoniae* when complement was active (*p* < 0.05) (Figures 6, 7, 8). Similar numbers of K5 and NHM *K. pneumoniae* were taken up by AGM PMBC and PMNL when opsonized with normal serum from seropositive and seronegative donors (Figures 6, 7, 8); however, significantly greater amounts of K1 were taken up by AGM PMBC when opsonized with normal serum from seronegative donors. Significantly lower amounts of K1 were taken up by PMNL when compared to other isolates, even in the presence of normal serum (*p* < 0.05) (Figures 6, 7, 8).

No oxidative burst activity in AGM PMNL and PBMC was detected after challenge with *K. pneumoniae* previously opsonized with normal serum or heat inactivated-serum from seropositive and seronegative donors. Stimulation of oxidative burst activity in AGM PMNL and PBMC with PMA, fMLP, pre-opsonized *E. coli* and TLR-2 (PAM_3_CSK_4_) resulted in positive detection when using the modified protocols of Gondwe et al. [22], using the PHAGOBURST kit (data not shown).

## Discussion

The components of the innate immune system, including humoral elements like complement, and cellular elements like PMNL and PBMC are critical to protect the host upon bacterial infection. Additionally, there is evidence of a role for both bactericidal and opsonizing antibody in immunity to members of the family Enterobactericeae [21, 22, 24]. On the other hand, bacterial pathogens acquire and employ different virulence factors upon contact with innate and adaptive immune system of the host to ensure colonization and dissemination within the host. Some of the best characterized virulence determinants of *K. pneumoniae* are the capsule, lipopolysaccharides, siderophores, and types 1 and 3 fimbriae [25]. As with other Gram-negative bacteria, the capsule is associated with attachment to host receptors, protection from phagocytosis, and with barrier function against innate host defense components such as complement, lysozyme, and oxidative mediated killing [26]. Distinct capsular components and an increased amount of capsular material in HMV *K. pneumoniae* have been described in hypervirulent *K. pneumoniae* human isolates [10]. However, little work elucidating the role of the HMV phenotype in *K. pneumoniae* pathogenicity exists, and no direct comparison of HMV and non-HMV isolates using components of the innate and adaptive immune system of naturally susceptible hosts has been performed. Here we examined the potential role of antibody and complement in the control of *K. pneumoniae* using in vitro assays. African green monkeys are not only extremely important animals in biomedical research, but also are naturally susceptible to *K. pneumoniae* infections. Due to their close phylogenetic relationship to humans, they are recognized as an intermediate animal model between humans and rodents, and are an indispensable model for human diseases [27, 28].

**Figure 9:**
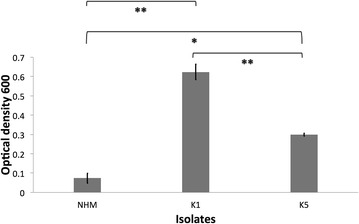
***Klebsiella pneumoniae*** **mucoviscosity levels.** Mucoviscosity of hypermucoviscous isolates K1, K5 and non-hypermucoviscous (NHM) was evaluated measuring optical density (OD)_600_ of normalized bacteria grown in LB broth after centrifugation. The error bars represent standard errors for triplicate samples, and the results shown are representative of three independent experiments. Asterisk indicates significant difference between treatments, *p* ≤ 0.05; Double asterisk indicates significant difference between treatments, *p* ≤ 0.001; Equal to indicates similarity between treatments.

The role of complement and antibodies and its interaction in conferring protection against klebsiellosis appears complex, particularly for HMV *K. pneumoniae*. In this study, HMV isolates (K1 and K5) were found to be resistant to complement-mediated killing as indicated by AGM serum and whole blood killing assays (Figures 4 and 5). Non-HMV isolates were at least partially susceptible to its effect as the removal of killing was observed in serum with heat inactivation of complement (Figures 4 and 5). Isolates K1 and K5 produced a significantly more mucoid capsule, than isolates designated non-HMV strains, and K1 was found more mucoid than K5 serotypes (*p* < 0.05) (Figure 9; Table 1). The biological significance of the greater mucoviscosity found in HMV K1 isolates when compared to HMV K5 isolates is unknown. The amount of capsular polysaccharides produced by *K. pneumoniae* is suspected to be significantly more important for resistance against complement than its chemical composition [29]; and together with lipopolysaccharide (LPS), these two virulence factors appear as major mechanisms of resistance to the bactericidal activity of complement [29–31]. Our results indicate similar resistance to complement by K1 and K5 HMV isolates (Figures 4 and 5); however, K5 were phagocytized by PBMC and PMNL) in greater amounts than K1 isolates in the presence of normal serum (*p* = 0.0006 and *p* = 0.0020, respectively) (Figures 6 and 7). Similar findings were reported by Cox et al. [17] were *khe*+, *rmpA*+, *magA*− isolates associated with the AGM PBMC to a greater extent than *khe*+, *rmpA*+, *magA*+), isolates (*p* < 0.05).

The complement system is an important component of innate immunity that consist of a cascade of soluble proteins and membrane-expressed receptors and regulators, which operates in plasma, tissues, cell surfaces and even within the cell. Complement (C3) activation can proceed via three enzymatic cascades, the classical, the alternative, and the mannose-binding lectin (MBL) pathways. The classical complement pathway is initiated by the binding of C1q to the Fc portion of antibody–antigen complexes on the bacterial surface, while the alternative and lectin pathways are activated by cell surface components of bacteria. Activation of any of these three pathways leads to the opsonization of the target with the complement component C3b. Formation of C3b by a set of programmed enzymatic reactions proceeds to the assembly of a membrane attack complex (MAC) and bacteriolysis. It also plays a central role in the inflammatory process and modulates the activity of T- and B-cells. In addition, the complement receptors present on phagocytes bind C3b and iC3b molecules on the bacteria to induce phagocytosis [24, 32]. Similar binding of C3/C3b and C5–9 was detected in NHM, K1 and K5 isolates (Figure 3); however as already mentioned, greater susceptibility to complement-mediated killing was found in NHM strains. Serum-resistant *K. pneumoniae* have a reduced ability to bind C1q, and this results in complement activation by serum-resistant strains principally via the alternative pathway; however serum-sensitive strains activate both the alternative and classical pathways, resulting in greater deposition of C3b and subsequent killing by the membrane attack complex [33, 34]. In this study, complement C3/C3b binding to *K. pneumoniae* was significantly greater when serum from seropositive donors was used (*p* < 0.05), and although not significantly different, there was slightly greater binding of C5-9 in serum from seropositive donors (Figure 3). However, whole blood and serum killing assays indicate greater survival of *K. pneumoniae* in the presence of normal serum from seropositive donors when compared with normal serum from seronegative animals. Therefore a distinct correlation between complement deposition and IgG titer supporting antibody-dependent complement-mediated killing as the mechanism of bactericidal activity was not found (Figures 4 and 5). In contrast to our findings, Yeh et al. [35] reported that K1 and K2 HMV *K. pneumoniae* isolates recovered from liver abscess showed resistance to serum from normal human patients, but were significantly more susceptible to killing effects of serum when incubated with serum from patients with recurrent K1 *K. pneumoniae* liver abscesses. However, a direct comparison to Yeh et al. [35] findings is not possible since it is unknown if our serum donors had mounted an antibody response to *K. pnemoniae* from sub-clinical, clinical or recurrent infection. In some cases, bacteria can resist complement-mediated killing by immune serum, and this phenomenon has been reported in other members of the family Enterobactericeae like *Salmonella enterica,* and other Gram-negative and Gram-positive pathogens [21, 22, 36, 37].

The role of anti-*Klebisella* antibodies as opsonins was also explored using AGM PBMC and PMNL in in vitro uptake assays. In these experiments, there was greater uptake of bacteria by PMNL and macrophages when bacteria was opsonized with normal serum, indicating a role of complement in opsonization (Figure 8); however the greater amount of IgG on groups designated as “seropositive donors” did not appear to be protective, and as indicated by whole blood killing assays, incubation in heparinized blood from seronegative donors resulted in significantly less survival of HMV and non-HMV isolates (*p* < 0.05). Additionally, no oxidative burst activity was detected in PMNL and PBMC incubated with opsonized bacteria. Similar results have been reported in human PMNL stimulated with *K. pneumoniae* serotype K1, K2, K4 and K5 [11, 38] Further work by Sahly et al. [39] identified that the lower response of respiratory burst in human PMNL produced by some *K. pneumoniae* could be associated with lack of manno(rhamno)biose in the capsular polysaccharides of this bacteria. Thus, although complement increased opsonization of all *K. pneumoniae* (Figure 8); HMV isolates appear to be resistant to killing even in presence of active complement and IgG and IgM. In *S. enterica*, there is a critical requirement for complement in opsonization and killing, as bacterium opsonization by antibodies alone results in negligible phagocytosis, oxidative burst and cellular killing by human peripheral blood cells [22]. In this study, significantly greater amounts of IgM were found binding to non-HMV isolates than to K1 and K5 isolates (Figure 1); thus IgM could be at least partially associated with greater protection at least against non-HMV isolates as indicated by greater susceptibility of non-HMV isolates in serum and whole blood killing. IgM has been shown to have more ability to activate complement on *S. enterica* than IgG [40]. The significantly greater amount of capsule present in HMV isolates K1 and K5 could be associated with complement mediated killing resistance in these isolates.

Immunoglobulin G is the most abundant immunoglobulin in primate serum. There are at least four different subclasses of IgG, and although they are highly conserved, they differ in their constant region, and have different effector functions [41]. It was interesting to find that seropositive donors had at least a partial deficiency in IgG2 (Figure 2). IgG2 deficiencies have been associated with increased susceptibility to certain bacterial infections [41]; and Alsaedi et al. [42] recently reported IgG2 deficiency and low IgM in a human patient presenting recurrent HMV *K. pneumoniae* bacteremia in the absence of pyogenic liver abscess or other localized metastatic *Klebsiella* infection.

A major limitation in this study was the low number of seropositive and seronegative AGM donors. In the future, a better understanding on the recurrence of *K. pneumoniae* in non-human primates and other animals, and complete characterization of immunogens recognized by the host could be use to investigate the protective capacity of specific immunoglobulins to klebsiellosis. This can result in novel vaccines for humans and animals. In previous work, K1 *K. pneumoniae* isolates that were opsonized with serum from patients with recurrent K1 *K. pneumoniae* liver abscesses presented a significant increase in PMNL phagocytosis, compared to rates in K1 bacteria opsonized with serum from normal patients [35]; thus it appears that primates are able to mount protective immunity to HMV isolates. Wu et al. [43] reported that anti-capsular polysaccharide monoclonal antibodies agglutinate different strains of *K. pneumoniae*, including K1 serotype, enhanced phagocytosis by human monocyte-derived macrophages and protected mice in laboratory controlled in vivo challenges with *magA*+ *K. pneumoniae.* Potential use of live-attenuated strains as vaccines has also been explored. Hsieh et al. [44] mutated O1:K1 and O1:K2 mutants deleting the O1 synthesizing genes and attenuated the strains as demonstrated in serum killing assays. Moreover, immunization of mice with a magA-mutant (K_1_^−^O_1_) against LPS O1 provided protection against infection with an O1:K2 strain, but not against infection with an O1:K1 strain. [44] Cleary, more research investigating the host-pathogen interaction of hypervirulent and HMV strains with different in vivo and in vitro models are needed to better understand the pathogenesis of this emergent pathogen and to develop efficacious vaccines for humans and other animals.

Our findings highlight the complexity of the non-human primate immune response to HMV isolates of *K. pneumoniae*. These isolates evaded important components of the AGM innate and adaptive immune system, including serum- mediated killing, PBMC and PMNL phagocytosis and respiratory burst. Deeper understanding of the pathophysiology of infection with HMV *K. pnemoniae* related to virulence, resistance, and predisposing AGM host factors is required.
